# Testing the effect of PAR1 inhibitors on *Plasmodium falciparum*-induced loss of endothelial cell barrier function

**DOI:** 10.12688/wellcomeopenres.15602.3

**Published:** 2020-07-07

**Authors:** Janet Storm, Yang Wu, Jill Davies, Christopher A. Moxon, Alister G. Craig

**Affiliations:** 1 Department of Tropical Disease Biology, Liverpool School of Tropical Medicine, Liverpool, L3 5QA, UK; 2 Wellcome Centre for Integrative Parasitology, Institute of Infection, Immunity and Inflammation, College of Medical Veterinary & Life Sciences, University of Glasgow, Glasgow, G12 8TA, UK; 3 Centre for Drugs and Diagnostics, Liverpool School of Tropical Medicine, Liverpool, L3 5QA, UK

**Keywords:** Plasmodium falciparum, cerebral malaria, PAR1, thrombin, endothelium, barrier function

## Abstract

**Background:** Sequestration and cytoadherence of *Plasmodium falciparum*-infected erythrocytes (IE) to microvascular endothelium alters endothelial barrier function and plays a role in the pathogenesis of severe malaria. Binding of IE is mediated by *P. falciparum* erythrocyte membrane protein 1 (PfEMP1) and the PfEMP1 variants that binds to endothelial protein C receptor (EPCR) have, in particular, been associated with the dysregulation of the coagulation/inflammation pathways in endothelial cells. This has prompted speculation about the role of protease-activated receptor-1 (PAR1) activation and signalling in causing endothelial activation and loss of barrier function in cerebral malaria.

**Methods: **We used a co-culture of primary human brain microvascular endothelial cells (HBMEC) with *P. falciparum* material, recombinant PfEMP1 or lysates from IE, and measured barrier function by trans endothelial electrical resistance (TEER).  A selection of PAR1 inhibitors was tested for their ability to reverse the *P. falciparum *and thrombin induced decrease in barrier function.

**Results:** An initial screen in the presence of recombinant PfEMP1 identified a few inhibitors that were able to reduce the rapid thrombin-induced barrier disruption even when activated protein C (aPC) was unable to do so. However, PAR1 inhibitors did not rescue the barrier dysfunction after co-culture with IE lysate.

**Conclusions:** The selected PAR1 inhibitors were able to reverse the disruption of barrier function by thrombin but did not reverse the IE lysate induced disruption of barrier function, implicating a different PAR1-independent mechanism.  These findings have implications for the design of adjunct therapies to reduce brain swelling in cerebral malaria.

## Introduction

**Figure 1.**
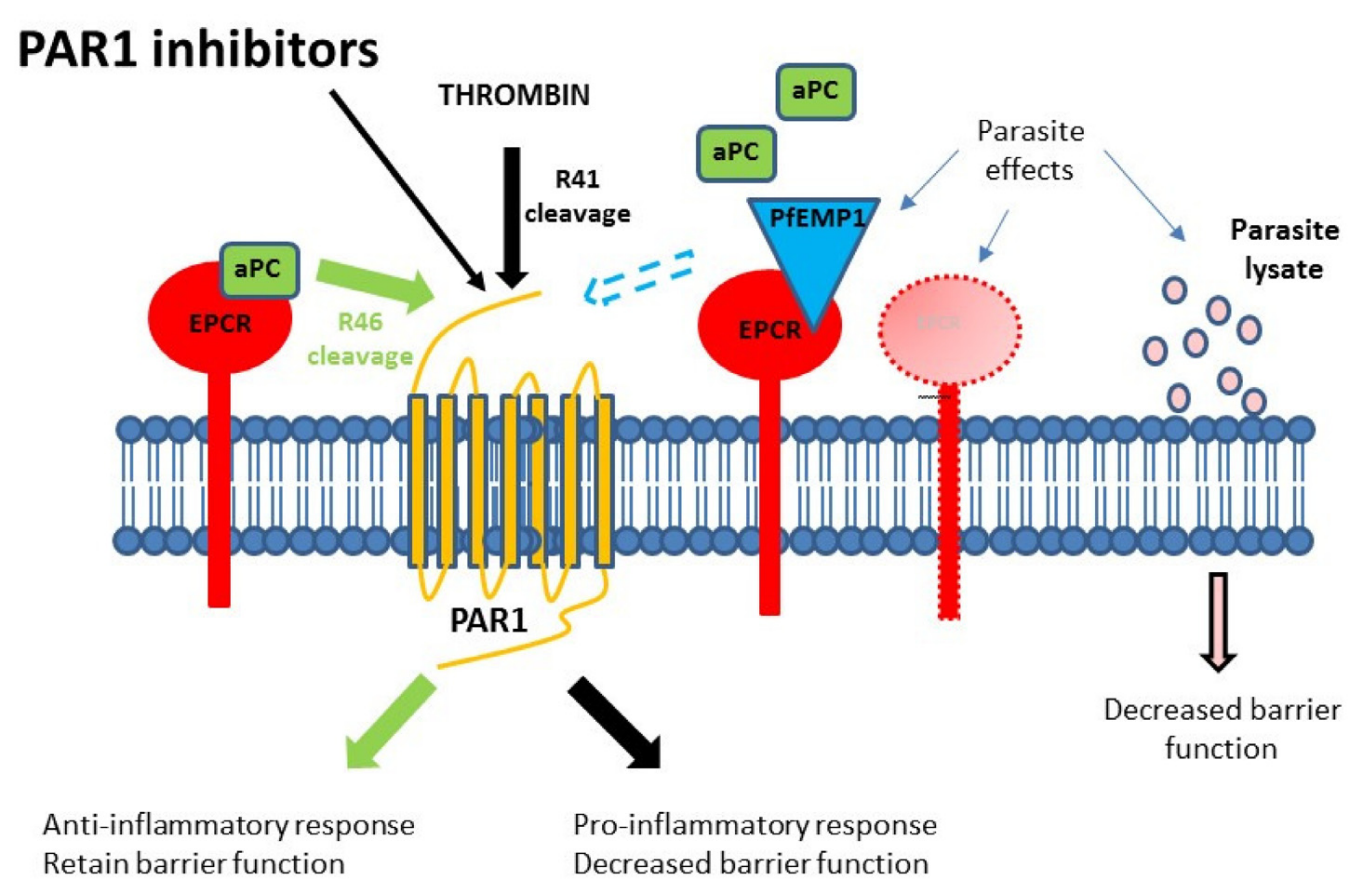
Schematic diagram of the proposed mechanisms of endothelial dysregulation via *Plasmodium falciparum* cytoadherence. Under normal circumstances, the presence of endothelial protein C receptor (EPCR)-activated protein C (aPC) on the endothelial surface is able to modulate the protease-activated receptor 1 (PAR1) response (by cleavage of R46) to thrombin along a cytoprotective pathway. In the presence of IE, the binding site for aPC can be occupied by *P. falciparum* erythrocyte membrane protein 1 (PfEMP1) or EPCR expression can be reduced, both limiting the availability of EPCR-aPC and resulting in cytopathic responses due to unmodified thrombin cleavage (R41) of PAR1. *P. falciparum*-infected erythrocytes (IE) are also able to release soluble factors that can influence endothelial integrity directly.

The pathology of cerebral malaria (CM) is not fully understood but is associated with sequestration of *P. falciparum-*infected erythrocytes (IE) and involves interactions between IE and host endothelial cells (EC) as well as host inflammation (for a review see ( Wassmer & Grau, 2017)). In a clinical study in Malawi, brain swelling was found to be strongly associated with fatal outcome in CM ( Seydel *et al.*, 2015), which has subsequently been confirmed by studies in India ( Mohanty *et al.*, 2017). The aetiology of this swelling is probably multi-factorial, involving cytopathic and vasogenic mechanisms (e.g. local and systemic inflammation; endothelial cell death; IE and erythrocyte accumulation). Multiple lines of work indicate involvement of the protein C (PC)–protease-activated receptor-1 (PAR1) axis and the consequences of the loss or inactivation of endothelial protein C receptor (EPCR) (for a review see ( Mohan Rao *et al.*, 2014)). PAR1 is a seven-transmembrane G protein-coupled receptor and is the prototypical thrombin receptor. It contains its own tethered ligand and exhibits biased antagonism. Cleavage events at two different sites lead to opposing effects: when cleaved at amino acid R41 by thrombin, PAR1 is pro-inflammatory and barrier disrupting whereas non-canonical cleavage of PAR1 at amino acid R46 by activated PC (aPC) has a barrier stabilising and anti-inflammatory effect, significantly reducing the inflammatory and barrier disruptive effect of thrombin on endothelial cells. Non-canonical cleavage of PAR1 by aPC is dependent on aPC binding to the active site of EPCR ( Figure 1). This dysregulation of the coagulation system does not often result in clinically evident thrombosis or bleeding in children and some researchers in this field have stressed the distinction between cytopathic and coagulant effects in this pathway ( Mosnier *et al.*, 2007). However, laboratory measurements of coagulation factors do show coagulation activation that is associated with fatal outcome in cerebral malaria (CM) ( Moxon *et al.*, 2015).

**Figure 2.**
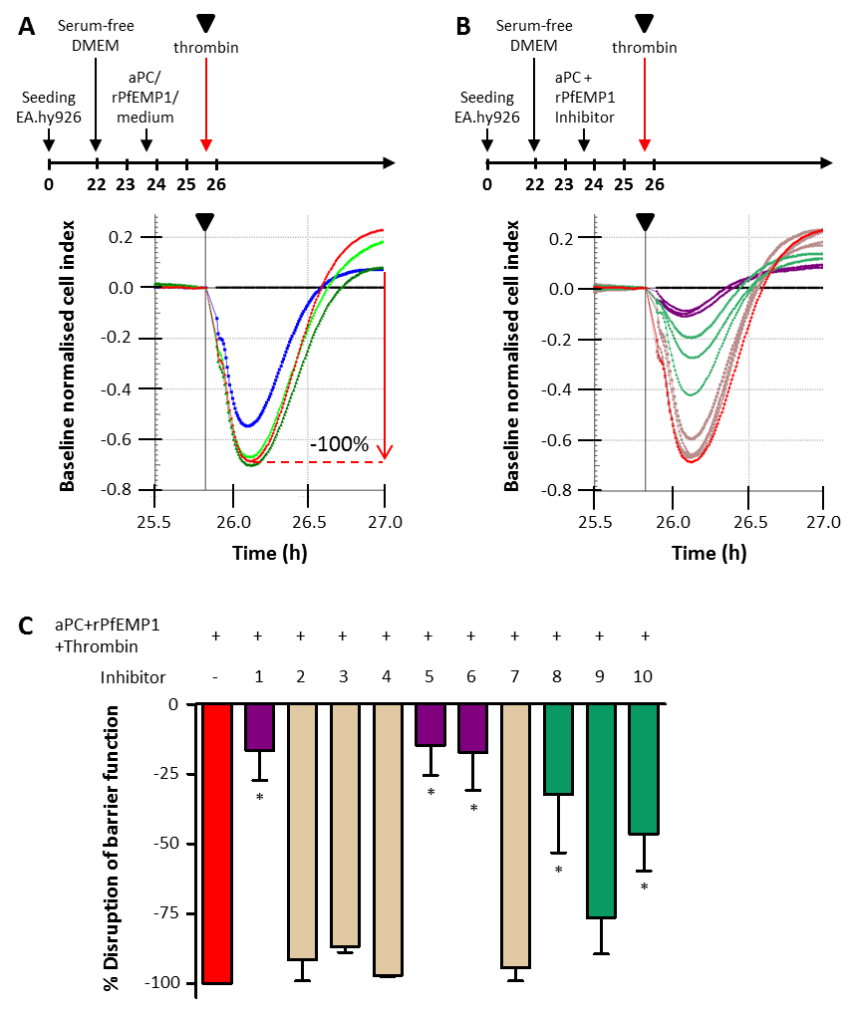
Measuring effect of protease-activated receptor 1 (PAR1) inhibitors on endothelial barrier function of EA.hy926 cells using trans endothelial electrical resistance. ( **A**) Experimental timeline and cell index traces for thrombin induced decrease in barrier function in EA.hy926 cells. Schematic of the experimental timeline (not to scale) indicating the addition of medium and modulators to the cells. Cell index was normalised at the time point immediately prior to the addition of 2 nM thrombin, indicated by the black triangle in the timeline and cell index trace, and medium (black line) was set as baseline. The decrease in normalised cell index by thrombin (red) was set at 100% and the effect of 57 nM aPC (blue), 25 nM recombinant *P. falciparum* erythrocyte membrane protein 1 (rPfEMP1) (bright green) and 57 nM activated protein C (aPC) and 25 nM rPfEMP1 combined (dark green) on thrombin induced decrease in barrier function was determined. ( **B**) The effect of inhibitors on thrombin induced decrease in barrier function. Inhibitors (concentrations in Table 1) were tested in the presence of 57 nM aPC and 25 nM rPfEMP1. The inhibitors in light brown do not have an effect, the inhibitors in light green have and intermediate effect and the inhibitors in purple reverse the effect of thrombin. ( **C**) Graph of the data depicted in B with corresponding colours. Thrombin induced decrease in barrier function was set as 100% (red) and shown are the mean ± SD of 3 independent experiments for the 10 inhibitors. *denotes a P-value <0.05.

Two mechanisms for the action of cytoadherence on coagulation/inflammation dysregulation have been proposed ( Figure 1). The direct binding of the *P. falciparum* variant surface protein *P. falciparum* erythrocyte membrane protein 1 (PfEMP1) to EPCR blocks the conversion of PC to aPC, affecting the ability of this host control system to control PAR1 cleavage by thrombin and altering the PAR1 signalling pathway ( Avril *et al.*, 2019; Gillrie *et al.*, 2015; Kessler *et al.*, 2017; Mosnier & Lavstsen, 2016; Sampath *et al.*, 2015). This mechanism is dependent on the presence of EPCR-binding parasite variants, and these PfEMP1 types are strongly associated with severe malaria ( Mkumbaye *et al.*, 2017; Storm *et al.*, 2019). However, the same effect can also be achieved through the reduction of expression of EPCR on the endothelial surface, which is thought to be expressed at lower levels in the brain microvasculature. We ( Moxon *et al.*, 2013) have shown that this does occur and is associated with CM, and while linked to IE sequestration, this appears to be due to receptor shedding rather than steric inhibition and does not require cytoadherence via EPCR ( Moxon *et al.*, 2013). Either mechanism would disrupt EPCR function and its capacity to form a complex with aPC and modify PAR1 signalling; thus leading to an unmodified effect of thrombin with pro-inflammatory changes and loss of EC barrier function. Because the dysfunction of EPCR is at the level of the receptor, these cannot be mitigated through the addition of aPC ( Figure 2A).

On embarking on this study, our hypothesis was that through both of these mechanisms, the Thrombin-PAR1 coagulation/inflammation axis plays a significant role in barrier loss in CM, and that adjunct treatments targeting this might alleviate mortality or post-CM neurological sequelae in CM. Since the activation of PC may be prevented by either of these mechanisms of EPCR disruption (steric inhibition or receptor cleavage) it is important to identify treatments that could be barrier stabilising to EPCR abrogation by either mechanism. We therefore investigated a range of PAR1 antagonists that are able to directly inhibit thrombin cleavage of the PAR1 extracellular domain without affecting other thrombin-dependent pathways. We tested their ability in preventing loss of barrier function in human endothelial cells in response to treatment with parasite material.

## Methods

### Culture of endothelial cells and *Plasmodium falciparum*

The human umbilical vein endothelial cell line EA.hy926 (ATCC) was cultured in DMEM medium supplemented with 10% foetal calf serum. Primary human brain microvascular endothelial cells (HBMEC, Cell Systems) were cultured in Endothelial Cell Growth Medium 2 containing 2% foetal calf serum, 5 ng/ml epidermal growth factor, 10 ng/ml basic fibroblast growth factor, 20 ng/ml insulin-like growth factor, 0.5 ng/ml vascular endothelial growth factor 165, 1 µg/ml ascorbic acid, 0.2 µg/ml hydrocortisone and 22.5 µg/ml heparin (EGM2, Promocell). The *Plasmodium falciparum* IT4 lab strains IT4var16 (ItG), IT4var14 (A4) and IT4var37 (4E12) were cultured according to our standard laboratory methods ( Wu *et al.*, 2018) in complete RPMI medium (RPMI 1640 with 25 mM HEPES, 11 mM glucose, 2 mM glutamine, 0.2% NaHCO _3_, 25 mg/l gentamicin and 10% Human Serum, pH 7.4) in normal group O red blood cells (RBC) at 3% haematocrit.

### rPfEMP1 preparation and PAR1 antagonists

Recombinant EPCR-binding PfEMP1 (rPfEMP1) was produced as previously described ( Turner *et al.*, 2015). In short, HIS-tagged cysteine-rich interdomain region domain α1 (CIDRα1) protein derived from the IT4var20 PfEMP1 sequence was produced in the baculo-virus expression system, purified by nickel affinity chromatography and validated for binding to EPCR in ELISA. PAR1 antagonists, supplied by Eisai Co. Ltd. (Japan), and listed in Table 1, or commercially sourced from Sigma (Varopaxar), were dissolved at 10 mM in DMSO and stored in aliquots at -20°C.

### Preparation of IE and IE lysates

Mid-late trophozoite stage IE were enriched by gelatin flotation and a suspension of 50% parasitaemia at 1% haematocrit in EGM2-min medium (EGM2 without hydrocortisone and heparin) was prepared. RBC were cultured overnight in complete RPMI medium and subjected to the same procedure as IE to obtain a suspension of 1% haematocrit.

Lysates of these IE and RBC suspensions were prepared by freeze-thawing three times. In TEER experiments, 300 µl IE suspension or lysate (1.5 × 10 ^7^ IE) was added per well (0.64 cm ^2^) in the E plate. In experiments to determine EPCR expression, 350 µl IE suspension or lysate (1.75 × 10 ^7^ IE) was added per well (1.9 cm ^2^) in a 24 well plate.

### Trans endothelial electrical resistance (TEER) analysis

Barrier function was measured by TEER with the iCELLigence™ system (ACEA Biosciences). EA.hy926 cells and HBMEC (up to passage 9) were seeded at 50,000 cells/cm ^2^ in 8-well E plates (L8, surface area 0.64 cm ^2^)) and TEER was recorded at specific time intervals and expressed as cell index. For the initial inhibitor screen, EAhy926 cells were grown overnight and when confluent, the culture medium was exchanged for serum- free DMEM medium and combinations of 25 nM rPfEMP1, 57 nM aPC or PAR1 inhibitors (see Table 1 for concentrations) or vehicle were added 2 hours prior to treatment with 2 nM thrombin (see Figure 2 for details, n=3). For subsequent experiments with HBMEC, the overnight culture medium was first replaced with EGM2 medium for 3 – 6 hours and then with EGM2-min medium 2 hours prior to the addition of 300 µl IE suspension (1.5 × 10 ^7^ IE per well) or 300 µl lysates in the absence or presence of selected PAR1 inhibitors (n=5). After ~20 hours, the additional effect of 2 nM thrombin was determined (n=2). Control experiments were performed with medium or inhibitor only. The cell index of medium only was set as baseline to correct for non-specific fluctuations of cell index during the experiment.

**Table 1.** Names of protease-activated receptor 1 inhibitors supplied by Eisai and concentrations used in the initial barrier disruption screen.

Inhibitor	Compound name	Conc. used
1	#91	0.1 µM
2	#33	2.5 µM
3	#01	2.0 µM
4	#62	5.0 µM
5	#04	0.1 µM
6	#39	0.5 µM
7	#38	1.0 µM
8	#11	0.1 µM
9	#96	1.0 µM
10	#90	0.5 µM

The decrease in barrier function by modulators was determined by normalising the cell index at the time point immediately prior to modulator addition. The maximum decrease in normalised cell index (NCI) by thrombin was determined for medium or lysate in the absence of inhibitor and set at 100%. The protective effect of inhibitor on the thrombin-induced disruption of barrier function calculated as follows:

∆NCI (thrombin + inhibitor)/∆NCI (thrombin − inhibitor) × 100.

For lysates, the normalised cell index was determined at 2 and 16 hours after adding the IT4var16 lysate. The decrease in normalised cell index was calculated and ∆NCI in absence of inhibitor was set at 100%. The effect of inhibitor on the lysate-induced decrease in barrier function was calculated as follows:

∆NCI (lysate + inhibitor)/∆NCI (lysate − inhibitor) × 100.

### Measuring EPCR expression on endothelial cells

The effect of IT4var14 and IT4var37 IE or IE lysate on the EPCR expression on HBMEC was determined after 16 – 20 hours of co-culture. A suspension of 50% parasitaemia at 1% haematocrit in EGM2-min medium was prepared and from this suspension lysate was also made, as described above. RBC and RBC lysate were used as control and 10 ng/ml TNF was used as a positive control, since it decreases EPCR expression. HBMEC were detached with Accutase ^®^, washed with cold PBS/1% BSA/ 2 mM EDTA, labelled with phycoerythrin conjugated rat anti-human EPCR antibody (Biolegend, 351904), washed and stained for cell viability with live/dead fixable yellow stain (Invitrogen). EPCR expression levels of viable cells were detected by flow cytometry.

### Statistical analysis

Statistical significance was calculated by ANOVA with Dunnett’s post-test comparing the mean value (± SD) for each individual modulator addition (inhibitor, IE, lysate or TNF) with the mean value (± SD) of the condition without modulator (GraphPad Prism version 5). A P-value < 0.05 was deemed significant.

## Results

### Thrombin-induced barrier disruption and the effect of prior treatment with either PAR1 inhibitors or aPC in the presence of rPfEMP1

**Figure 3.**
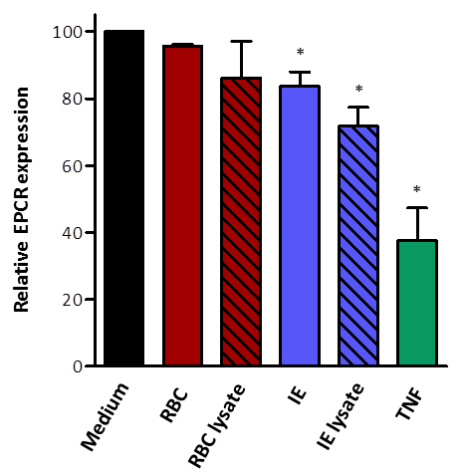
Endothelial protein C receptor (EPCR) expression on human brain microvascular endothelial cells after overnight culturing with *P. falciparum*-infected erythrocytes (IE) or IE lysate. EPCR expression was determined by flow cytometry after overnight culturing with IT4var14 or IT4var37 IE or IE lysate (n=2). Red blood cells (RBC), RBC lysate and tumour necrosis factor (TNF) were included as control. EPCR expression on cells with medium only was set at 100%. Shown is mean ± SD of the combined IT4var14 and IT4var37 data (n=4), RBC data (n=2) and the TNF data (n=2) with * indicating a P-value <0.05.

In the initial screen we investigated whether the effect of EPCR binding by PfEMP1 on thrombin-mediated barrier disruption of the human umbilical vein endothelial cell line EA.hy926 could be modified by PAR1 inhibitors. The loss of barrier function caused by the addition of thrombin was restored by the prior addition of aPC in the absence of rPfEMP1. However, aPC had no effect on preventing loss of barrier function when rPfEMP1 was bound to EPCR (see condition rPfEMP1-APC-thrombin in Figure 2; note that neither aPC nor rPfEMP1 treatment alone had significant effects on EC barrier function) blocking aPC-mediated barrier strengthening. Thus, aPC might not be a useful treatment when EPCR-binding IE are present, such as in severe malaria. However, normal barrier function was obtained with the addition of inhibitors 1, 5 and 6 ( Figure 2), suggesting that they are able to act to stabilise EC barrier function despite the loss of protective aPC-mediated PAR1 cleavage. Raw data for Figure 2, in addition to that for Figure 3– Figure 6, are available as Underlying data ( Storm *et al.*, 2020).

**Figure 4.**
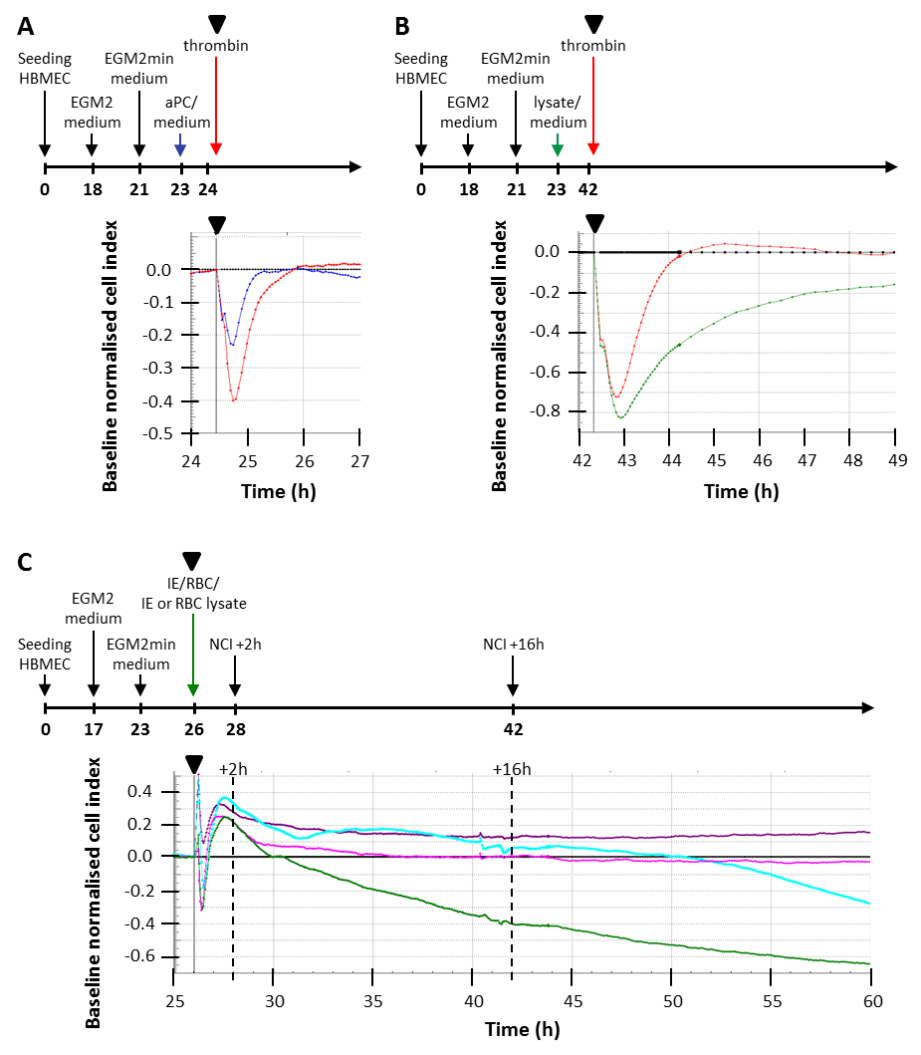
Measuring endothelial barrier function of human brain microvascular endothelial cells (HBMEC) using trans endothelial electrical resistance. ( **A**) Experimental timeline and cell index traces for thrombin induced decrease in barrier function in HBMEC. Schematic of the experimental timeline (not to scale) indicating the addition of medium and modulators to HBMEC. Cell index was normalised at the time point immediately prior to the addition of 2 nM thrombin, indicated by the black triangle in the timeline and cell index trace, and medium (black line) was set as baseline. Shown are the effect of 2 nM thrombin (red) on barrier function and the protection by 10 nM activated protein C (aPC) (blue) added 1 hour prior to thrombin. ( **B**) Schematic of the experimental timeline and cell index traces for thrombin induced decrease of barrier function after lysate exposure and its recovery over 7 hours in HBMEC. Cell index was normalised at the time point immediately prior to the addition of 2 nM thrombin, indicated by the black triangle in the timeline and cell index trace, and medium (black line) was set as baseline. Shown are the effects of 2 nM thrombin on barrier function of cells in medium (red) and cells exposed to *P. falciparum* infected erythrocytes (IE) lysate for 18.5 hours (green). ( **C**) Decrease in barrier function by IE, RBC and their lysates. Cell index of HBMEC was monitored and normalised at the time point immediately prior to the addition of cells or lysates, indicated by the black triangle at 26 hours in the timeline and cell index trace. Medium only was set as baseline (black line). Normalised cell index is shown for 35 hours for red blood cells (RBC) (purple), RBC lysate (magenta), ** IE (cyan) and IE lysate (green). The decrease in normalised cell index was measured between 2 and 16 hours after addition of cells or lysate.

**Figure 5.**
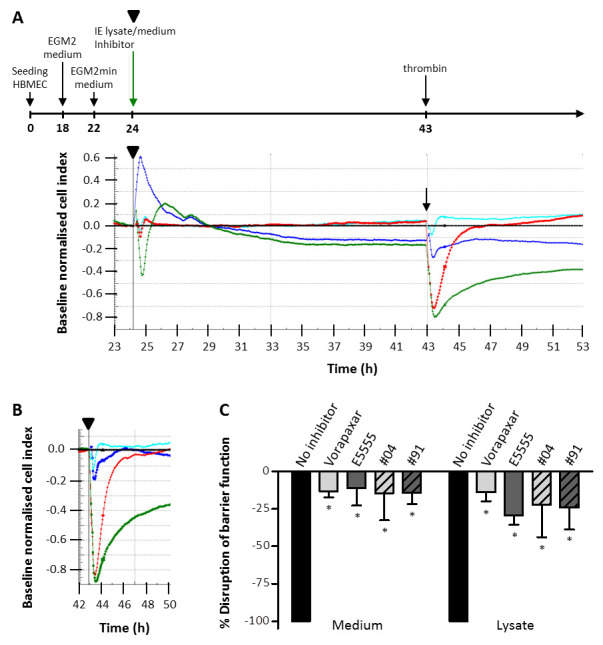
Effect of inhibitors on thrombin-induced decrease in barrier function of human brain microvascular endothelial cells (HBMEC). ( **A**) Experimental timeline and representative cell index traces for *P. falciparum* infected erythrocyte (IE) lysate induced decrease in barrier function in HBMEC and the effect of 0.3 µM Vorapaxar. Schematic of the experimental timeline (not to scale) indicating the addition of IE lysate and Varopaxar. Cell index traces are shown for IE lysate in the absence (green) and presence of Varopaxar (blue) and medium in the absence (red) and presence of Varopaxar (cyan). Cell index was normalised at the time point immediately prior to the addition of IE lysate, indicated by the black triangle in the timeline and cell index trace, and medium (black line) was set as baseline. After 19 hours 2 nM thrombin was added as indicated by the arrow. ( **B**) The same cell index traces as in **A**, but normalised at the time point prior to the addition of thrombin (black triangle). Vorapaxar (blue and cyan) reverses the effect of thrombin. ( **C**) Graph of the inhibitor data analysed as depicted in **B**. The maximum decrease in normalised cell index by thrombin was determined for medium or lysate in the absence of inhibitor and set at 100%. Shown are the mean ± SD of 2 independent experiments, with * indicating a P-value <0.05 compared to no inhibitor.

**Figure 6.**
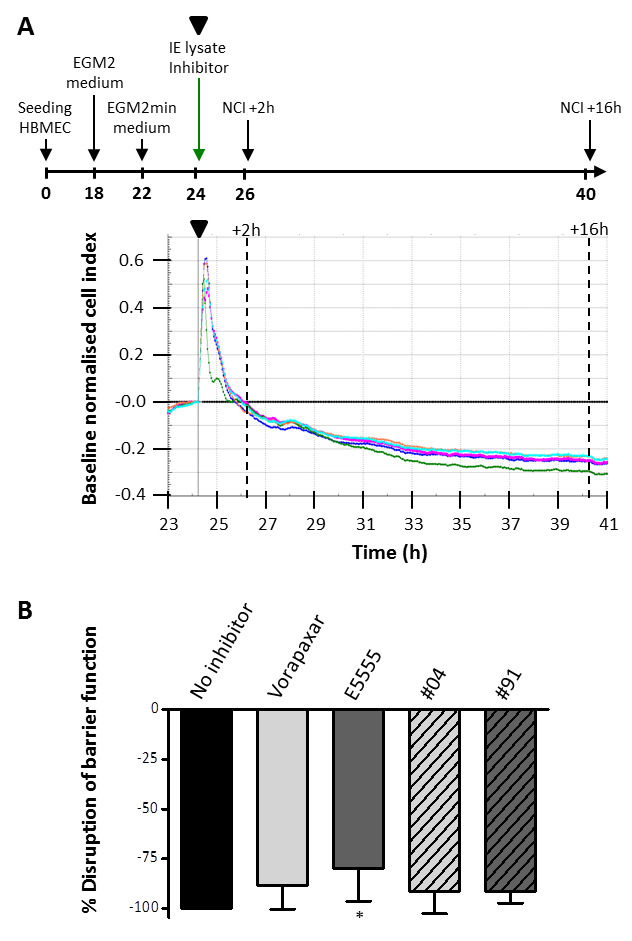
Effect of inhibitors on lysate-induced decrease in barrier function of human brain microvascular endothelial cells (HBMEC). ( **A**) Experimental timeline and cell index traces for *P. falciparum* infected erythrocyte (IE) lysate induced decrease in barrier function in HBMEC and the effect of the four inhibitors (0.3 µM). Schematic of the experimental timeline (not to scale) indicating the addition of IE lysate in the absence and presence of inhibitor to the cells. Cell index was normalised at the time point immediately prior to the addition of IE lysate, indicated by the black triangle in the timeline and cell index trace, and medium (black line) was set as baseline. The normalised cell index was determined at 2 and 16 hours after adding the lysate in the absence of inhibitor (green) or in the presence of Vorapaxar (blue), E5555 (magenta), #04 (cyan) or #91 (coral). ( **B**) Graph of the data depicted in A. The decrease in normalised cell index between 2 and 16 hours was calculated and this ∆normalised cell index in absence of inhibitor was set at 100%. Shown is the mean ± SD of 5 independent experiments with * indicating a P-value <0.05 compared to no inhibitor.

### Developing an endothelial cell barrier model using IE lysates

Based on these findings we identified a subset of the Eisai compounds that were able to inhibit the action of thrombin on barrier function in the presence of rPfEMP1 and aPC. Next we used a co-culture system with primary HBMEC and IE. The work focussed on three Eisai compounds: E5555 (Atopaxar ( Serebruany *et al.*, 2009)), #04 (inhibitor 5) and #91 (inhibitor 1). E5555 was included as it has been through Phase 1 and Phase 2 clinical testing. In addition, we used the clinically licenced PAR1 inhibitor Vorapaxar (brand name Zontivity (Aralez Pharmaceuticals)), obtained as a non-clinical compound from Sigma-Aldrich.

The effect of PfEMP1 ligation to EPCR on barrier function has been described elsewhere ( Gillrie *et al.*, 2015; Kessler *et al.*, 2017), but there has been little work on the impact of EPCR removal from the endothelial surface during cytoadherence. To address this, we focussed on EPCR abrogation in our co-culture model and first measured the EPCR expression on HBMEC after overnight co-culture with RBC, RBC lysate, IE or IE-lysate ( Figure 3). RBC and RBC lysate did not significantly reduce the EPCR expression and IE lysates had a more pronounced effect on EPCR expression compared to whole IE. Lysates were easier to prepare and more reproducible in TEER experiments, we therefore switched to a HBMEC/IE lysate co-culture system for subsequent experiments.

We observed that there were two ‘behaviours’ taking place in the TEER system ( Figure 4):

- A. A rapid response to thrombin in reducing EC barrier function transiently, which could be controlled under normal conditions using aPC ( Figure 4A).
- B. A slower reduction in barrier function, activated by treatment with IE lysate and producing a cumulative effect over 30 hours ( Figure 4C) that was not seen in control wells with RBC lysate where barrier function remained constant throughout the incubation period. IE had less of an effect on barrier function than IE lysate. The fluctuations in cell index in the first 2 hours are consistent in each experiment, probably due to the addition of cells or cellular material. After exposure to IE lysate, barrier function could still be further decreased by thrombin, but recovery was slow and in some cases barrier integrity was not restored to the level prior to thrombin addition ( Figure 4B).

Behaviour A was as expected and confirms that aPC rescues thrombin induced barrier disruption in HBMEC as seen above in EAhy926 cells. Behaviour B has been reported previously by several investigators using a range of co-culture systems ( Tripathi *et al.*, 2007) ( Gillrie *et al.*, 2012) ( Avril *et al*., 2019), with either IE or parasite material affecting barrier integrity. The delayed recovery after thrombin addition was also reported by Avril *et al*. when using schizont-stage IE in their TEER experiments ( Avril *et al*., 2019). Based on these findings we have tested the compounds for their ability to block both behaviours.

### Effect of PAR1 inhibitors on thrombin-induced and lysate-induced reduction in EC barrier function

Compounds were tested for their ability to inhibit disruption of barrier function by thrombin and the slower effect caused by the IE lysate directly. All tested PAR1 inhibitors were able to significantly reverse thrombin-induced barrier disruption even in the presence of IE lysate ( Figure 5). However, three of the four inhibitors had no significant effect on the lysate-induced decrease in barrier function when used at 0.3 µM ( Figure 6) or at 0.6 µM (see *Underlying data*; Storm *et al.*, 2020). Only E5555 significantly reverses the decrease in barrier function by IE lysate (-79.9 ± 16.5%, P = 0.026), albeit only by 20%. This still leaves a substantial EC barrier disruption by IE lysates, indicating that this phenomenon is independent of PAR1.

## Discussion

There is considerable interest in developing adjunct therapies for CM based on controlling brain swelling, following the MRI finding of a strong association between this phenotype and death from CM ( Mohanty *et al.*, 2017; Seydel *et al.*, 2015) and that brain swelling has also been associated with EPCR-binding IE phenotype ( Kessler *et al.*, 2017). As expected, the PAR1 inhibitors (including Vorapaxar) were able to block the effect of thrombin on transient reduction in barrier function, including in the presence of IE lysate. However, they had no significant effect on the slower reduction in barrier function caused by the lysate itself (thrombin independent).

Blocking of the thrombin-mediated pathway with the PAR1 antagonists might reduce brain swelling sufficiently to relieve mortality/morbidity in CM, but whether these types of inhibitors could be used safely in children/adults with multiple ring haemorrhages in their brains is a concern given the potential bleeding risk, owing to their inhibition of PAR1 in platelets ( https://www.rxlist.com/zontivity-side-effects-drug-center.htm). Further consideration of risk/benefit calculations will be required to answer this.

The slower effect on brain barrier function by IE lysate requires further investigation. Other work has implicated a role for β-catenin in regulating brain barrier integrity ( Gallego-Delgado *et al.*, 2016), showing that IE-derived soluble mediators are able to cause a reduction in TEER of brain endothelial cells over several hours. Similarly, parasite kinins were also shown to reduced brain endothelial cell barrier function, as well as enhancing IE cytoadherence ( Silva *et al.*, 2019), and HRP2 has been implicated in modifying barrier function ( Pal *et al.*, 2016). Parasite histones have also been shown to cause barrier disruption on lung and brain endothelial cells and to be released in culture by IE and *in vivo* in patients with CM ( Gillrie *et al.*, 2012) and are increased in children with CM and associated with blood brain barrier breakdown and brain swelling ( Moxon *et al.*, 2019). Despite the lysate induced decrease in barrier function, thrombin could still reduce the barrier function even further ( Figure 4B and Figure 5A). However, the recovery was delayed, an observation that was also reported in TEER experiments with HBMEC and schizont-stage IE, but not with trophozoite-stage IE ( Avril *et al.*, 2019). It is likely that that schizont-stage IE rupture during co-culture and release mediators, causing similar effects to IE lysate. We do not know whether the concentrations of parasite factors used in our model to cause endothelial barrier breakdown are the same as those seen during a malaria infection, and measurements from patient plasma may only partially reflect the local concentrations of these in vessels with high levels of cytoadherent IE. New dynamic 3D models of cytoadherence may be able to resolve this issue.

For histones, there are some therapeutic avenues that could be explored. Non-anticoagulant heparins prevent histone-induced lethality in bacterial sepsis models *in vivo* ( Wildhagen *et al.*, 2014) and block the toxic effects of plasmodial histones *in vitro*. A non-anticoagulant heparin, Sevuparin, is being used in phase II trials for malaria for its potential to block parasite binding ( Leitgeb *et al.*, 2017; Saiwaew *et al.*, 2017). Based on the work in this paper, an option would be to consider a combination therapy, based on stopping the thrombin-mediated effect using a PAR1 inhibitor combined with a non-anti-coagulant heparin to mitigate the histone effect. Interestingly, the two pathways are not completely separate, as aPC cleaves histones to regulate histone concentration in the vascular compartment ( Gillrie *et al.*, 2012; Xu *et al.*, 2009) and histones can reduce aPC production ( Kowalska *et al.*, 2014).

In summary, we have shown that PAR1 inhibitors are able to reduce thrombin-induced barrier disruption even when the normal aPC control pathway has been disabled due to direct EPCR engagement with PfEMP1 or by reduced EPCR expression on EC induced by parasite lysates. The identification of a second pathway for barrier disruption induced by parasite lysate that is insensitive to PAR1 inhibitor treatment suggests that a more complex approach to adjunct therapy may be needed.

## Data availability

### Underlying data

Figshare: Data acquired from testing the effect of PAR1 inhibitors on Plasmodium falciparum-induced loss of endothelial cell barrier function. https://doi.org/10.6084/m9.figshare.11558889 ( Storm *et al.*, 2020).

This project contains the following underlying data:

- Combined analysis data for fig 2_TEER_includes normalisation.csv (normalised data used to generate Figure 2).
- Combined analysis data for fig5_TEER_includes normalisation.csv (normalised data used to generate Figure 5).
- Combined analysis data for fig6_TEER_includes normalisation.csv (normalised data used to generate Figure 6).
- Data for fig2A+B+C_TEER_EA cells+inhibitors+thrombin_3.csv.
- Data for fig2C_TEER_EA cells+inhibitors+thrombin_1.csv.
- Data for fig2C_TEER_EA cells+inhibitors+thrombin_2.csv.
- Data for fig3_flow cytometry HBMEC.csv.
- Data for fig4A_TEER_aPC+thrombin_all time points shown.csv.
- Data for fig4B_TEER_RBC+IE+lysate_all time points shown.csv.
- Data for fig5+6_TEER_lysate+thrombin_#04-2+#91-2.csv.
- Data for fig5+6_TEER_lysate+thrombin_#91-1.csv.
- Data for fig5+6_TEER_lysate+thrombin_E5555-1+#04-1.csv.
- Data for fig5+6_TEER_lysate+thrombin_E5555-2.csv
- Data for fig5+6_TEER_lysate+thrombin_Vorapaxar-1.csv.
- Data for fig5+6_TEER_lysate+thrombin_Vorapaxar-2.csv.
- Data for fig6_TEER_lysate_Vorapaxar+E5555+#04+#91-1.csv.
- Data for fig6_TEER_lysate_Vorapaxar+E5555+#04+#91-2.csv.
- Data for fig6_TEER_lysate_Vorapaxar+E5555+#04+#91-3.csv.

Data are available under the terms of the Creative Commons Attribution 4.0 International license (CC-BY 4.0).

## Author information

Janet Storm and Yang Wu are joint first authors; Christopher A. Moxon and Alister G. Craig are joint last authors.
