# Supplementary material for: Testing the effect of PAR1 inhibitors on Plasmodium falciparum-induced loss of endothelial cell barrier function
Source: Wellcome Open Res. 2020 Jul 7;5:34. Originally published 2020 Feb 19. [Version 3] doi: 10.12688/wellcomeopenres.15602.3 (PMC7364184; doi:10.12688/wellcomeopenres.15602.3)
Supplement: Peer review history

# Peer review: Reviewer response for version 3

**DOI:** 10.21956/wellcomeopenres.17712.r39456

**Published:** 2020-07-15

**Authors:** Ana Rodriguez

**Affiliations:** 1 Department of Microbiology, New York University, New York, NY, USA

I have revised their response and everything is fine.

Is the work clearly and accurately presented and does it cite the current literature?

Yes

If applicable, is the statistical analysis and its interpretation appropriate?

Yes

Are all the source data underlying the results available to ensure full reproducibility?

Yes

Is the study design appropriate and is the work technically sound?

Yes

Are the conclusions drawn adequately supported by the results?

Yes

Are sufficient details of methods and analysis provided to allow replication by others?

Partly

Reviewer Expertise:

NA

I confirm that I have read this submission and believe that I have an appropriate level of expertise to confirm that it is of an acceptable scientific standard.

# Peer review: Reviewer response for version 2

**DOI:** 10.21956/wellcomeopenres.17537.r38878

**Published:** 2020-06-05

**Authors:** Ana Rodriguez

**Affiliations:** 1 Department of Microbiology, New York University, New York, NY, USA

In the methods, Preparation of IE and IE lysates, the concentration of lysate that is added to the endothelial cells is still missing. This can be expressed as number of lysed IE per surface area of the well. This measurement is important since it provides an idea of the likelihood that this phenomenon could be taking place i *n vivo.*

Is the work clearly and accurately presented and does it cite the current literature?

Yes

If applicable, is the statistical analysis and its interpretation appropriate?

Yes

Are all the source data underlying the results available to ensure full reproducibility?

Yes

Is the study design appropriate and is the work technically sound?

Yes

Are the conclusions drawn adequately supported by the results?

Yes

Are sufficient details of methods and analysis provided to allow replication by others?

Partly

Reviewer Expertise:

Malaria, cerebral malaria, malaria anemia, malaria pathogenesis

I confirm that I have read this submission and believe that I have an appropriate level of expertise to confirm that it is of an acceptable scientific standard, however I have significant reservations, as outlined above.

# Response

**Published:** 2020-06-10

**Authors:** Janet Storm

**Affiliations:** Liverpool School of Tropical Medicine, UK

The concentration of IE added to HBMEC was added to the methods describing the TEER experiments in version 2:

“For subsequent experiments with HBMEC, the overnight culture medium was first replaced with EGM2 medium for 3 – 6 hours and then with EGM2-min medium 2 hours prior to the addition of 300 µl IE suspension (1.5 x 10 ^7^ IE per well) or 300 µl lysates”

The surface area of the wells and seeding density of HBMEC is also provided in the same methods section. Lysates were prepared from these IE suspensions, so would be an equivalent amount.

# Peer review: Reviewer response for version 1

**DOI:** 10.21956/wellcomeopenres.17089.r38475

**Published:** 2020-04-20

**Authors:** Ana Rodriguez

**Affiliations:** 1 Department of Microbiology, New York University, New York, NY, USA

This work explores the possible mechanisms underlying cerebral malaria pathogenesis. It is therefore very relevant. The authors develop an *in vitro* model of the BBB and test the effect of *Plasmodium falciparum* and its effect on the ePCR/thrombin/PAR1 axis in barrier function of brain endothelial cells. The findings implicate that the parasite causes a more potent delayed barrier disruption that is not related to the classical ePCR/PAR1 model.

 Minor points:

- Include the number of IE per surface area added in every experiment or the equivalent for IE lysates.
- Include the number of cells seeded per well (or surface area) used in xCelligence experiments.
- Include the Cell index of cultures after the overnight growth before starting the experiments.
- Indicate the stage of IE used in the experiments or if asynchronous cultures were used. If a single stage was used, explain how was it isolated.
- "For subsequent experiments with HBMEC, the overnight culture medium was first replaced **with EGM2 medium** and then with EGM2-min medium 2 hours prior to the addition of lysates in the absence or presence of selected PAR1 inhibitors (n=5)." please indicate the time of incubation with EGM2 medium.
- In Fig. 2, the effect of aPC and recombinant PfEMP1 alone on cells should be included as controls.
- In Fig. 3, a control of uninfected RBC and their lysate should be included.
- In Fig.4, it is not clear which are the lines corresponding to lighter and darker shades of green. Please use different colors.
- In Figure 5, it would be interesting to see the original cell index graph showing the effect of thrombin on cells already incubated with the parasite lysate for 20 h.
- In Figure 6 it is stated that "the normalised cell index was determined at 2 and 16 hours", but it is not clear which time point is shown in the graph or if it is the different between the values at these two time points. It would be informative to see the original cell index graphs as in figure 4 or to show the results at 2 h and 16 h separately in two independent graphs.

Is the work clearly and accurately presented and does it cite the current literature?

Yes

If applicable, is the statistical analysis and its interpretation appropriate?

Yes

Are all the source data underlying the results available to ensure full reproducibility?

Yes

Is the study design appropriate and is the work technically sound?

Yes

Are the conclusions drawn adequately supported by the results?

Yes

Are sufficient details of methods and analysis provided to allow replication by others?

Partly

Reviewer Expertise:

Malaria, cerebral malaria, malaria anemia, malaria pathogenesis

I confirm that I have read this submission and believe that I have an appropriate level of expertise to confirm that it is of an acceptable scientific standard, however I have significant reservations, as outlined above.

# Response

**Published:** 2020-05-19

**Authors:** Janet Storm

**Affiliations:** Liverpool School of Tropical Medicine, UK

We thank the reviewer for her constructive comments and have addressed these in our reply and in the manuscript.

**Include the number of IE per surface area added in every experiment or the equivalent for IE lysates.**

For IE and IE lysates, 300 µl was added per well and always at 50% parasitaemia at 1% haematocrit. This is 1.5 x 10 ^7^ IE per well (0.64 cm ^2^ surface area). This is now added to the methods. In addition, related to comment 4, the preparation of IE suspension has also been added to the methods section.

**Include the number of cells seeded per well (or surface area) used in xCelligence experiments**.

Cell were seeded at 50,000 cells/cm ^2^, equal to 32,000 cells/well (surface area per well is 0.64 cm ^2^). This is added to the methods.

**Include the Cell index of cultures after the overnight growth before starting the experiments.**

On average the cell index, after refreshing the medium and before adding lysates and/or inhibitors, is 6.9. This information is available in full in the raw data ( https://doi.org/10.6084/m9.figshare.11558889).

**Indicate the stage of IE used in the experiments or if asynchronous cultures were used. If a single stage was used, explain how was it isolated.**

The section in the methods describing the preparation of lysate has been altered and includes the preparation of IE suspension at 50% parasitaemia at 1% haematocrit. Lysates were made from these IE suspensions.

**"For subsequent experiments with HBMEC, the overnight culture medium was first replaced with EGM2 medium and then with EGM2-min medium 2 hours prior to the addition of lysates in the absence or presence of selected PAR1 inhibitors (n=5)." please indicate the time of incubation with EGM2 medium.**

Fresh EGM2 was normally added in the morning, resulting in 3-6 hrs incubation. This has been added to the methods.

**In Fig. 2, the effect of aPC and recombinant PfEMP1 alone on cells should be included as controls.**

The TEER traces in figure 2A are a representative and are derived from an experiment that did not include aPC and rPfEMP1 on their own. We choose this representative experiment as it included the inhibitor data shown in figure 2B. From other experiments it was clear that neither aPC nor rPfEMP1 affected the normalised cell index more that 5%, this has been added to the results section.

**In Fig. 3, a control of uninfected RBC and their lysate should be included.**

These controls are now added to figure 3, its legend and the text describing the result.

**In Fig.4, it is not clear which are the lines corresponding to lighter and darker shades of green. Please use different colors.**

The colours in new figure 4C (was 4B) have been changed. IE lysate is now green and IE is cyan.

**In Figure 5, it would be interesting to see the original cell index graph showing the effect of thrombin on cells already incubated with the parasite lysate for 20 h.**

Cell index traces have been added to figure 5, as requested by the other reviewer too. It shows the slow recovery from thrombin after co-culture with IE lysate. For clarity, a similar cell index trace has been added to figure 4.  See also our reply to the other reviewer.

**In Figure 6 it is stated that "the normalised cell index was determined at 2 and 16 hours", but it is not clear which time point is shown in the graph or if it is the different between the values at these two time points. It would be informative to see the original cell index graphs as in figure 4 or to show the results at 2 h and 16 h separately in two independent graphs.**

Representative cell index traces have also been added to figure 6 and indicate the 2 and 16 hour time points. The difference between the normalised cell index at these time points was calculated and used to make the graph. These calculations are also provided in the raw data file “Combined analysis data for fig6_TEER_includes normalisation_2020-02-04.csv”.

# Peer review: Reviewer response for version 1

**DOI:** 10.21956/wellcomeopenres.17089.r37975

**Published:** 2020-03-06

**Authors:** Maria Bernabeu

**Affiliations:** 1 European Molecular Biology Laboratory, Barcelona, Spain

The study from Storm *et al.*, describe how PAR1 inhibitors could counteract the loss of endothelial barrier that occurs in cerebral malaria. The authors show that PAR1 inhibitors revert barrier disruption mediated by thrombin, but are not effective against long-term barrier breakdown caused by parasite lysates. The finding that PAR1 inhibitors are, even more effective than aPC, at rescuing thrombin-induced barrier opening  in the presence of PfEMP1 EPCR binders is important for the field. The finding that other therapeutic approaches will be needed for barrier caused by parasitic molecules/toxins is also relevant. Overall, the study is well-designed and the conclusions are supported by the results. However, the study might benefit from additional improvements.

**Major comments:**

 Methods:

- The authors used the same parasitemia and hematocrit for long-term culture or to prepare the IEs lysate and they obtained significantly differences in barrier dysfunction. Did the authors observe merozoite egress after long term IE incubation? Are the molecules released on a mid-trophozoite lysate a good proxy of schizont molecules released after rupture? Is the concentration used “physiologically” present in the cerebral malaria microvasculature. Although I agree that the concentration used in this study might be necessary to observe a major barrier breakdown in *in vitro *cultures, it might not be accurately mimic the pathogenic mechanisms on patients. A sentence in the discussion reflecting the limitations of the model might improve the interpretation of the paper by readers outside the field.

 

 Results:

-  Long term incubation with both IE or IE lysate showed 1) reduced surface expression of EPCR and reduced HBMEC cell index. However, the reduced cell index might not be a direct consequence of loss of EPCR in the surface. As the authors accurately describe in the discussion, loss of barrier in their experiments is more likely a consequence of the presence of parasite histones, HRP2 or other soluble mediators. However, the hypothesis exposed in the introduction might be misleading for the average reader. *“Our hypothesis is that, through both of these mechanisms, the Thrombin-PAR1 coagulation/inflammation axis plays a significant role in barrier loss in CM, and that adjunct treatments targeting this might alleviate mortality or post-CM neurological sequelae in CM. Since the activation of PC may be prevented by either of these mechanisms of EPCR disruption (steric inhibition or receptor cleavage) it is important to identify treatments that could be barrier stabilising to EPCR abrogation by either mechanism.”* Since we don’t understand quite well the molecular mechanisms that induce loss of endothelial integrity and surface EPCR, the authors should be more careful while establishing associations.
- I would recommend two include both 1) representative traces of cell index and bar plots in figure 3, 4, 5 and 6 in a similar way than in figure 2. Or include it in supplementary data. Was the experiment shown in figure 4 done on independent days? Although TEER measurements are highly variable depending on the day of the experiment, box plots and dot plots might better represent data distribution than bar graphs.

Discussion:

- Avril *et al.*, 2019 showed using the same methodology that schizont-stage parasites (EPCR binding strains) prolonged thrombin-induced barrier disruption in resting and activated HBMEC, and HBMEC do not get back to baseline after IE incubation. Thrombin and IE colocalization is frequent in the microvasculature in cerebral malaria autopsies. As the effects seen in Avril *et al.* are likely to occur in patients this experiment is highly relevant. Is the experiment in Figure 5 equivalent to Avril *et al.*, 2019 but with trophozoites lysates expressing CD36-binding PfEMP1? At which time-scale was the CI measured in figure 5? A representative cell index trace in Figure 5 will clarify that.

 

**Minor comments:**

 Abstract:

- “However, in the IE lysate co-culture system we identified a mechanism that slowly reduces barrier function and which is insensitive to PAR1 inhibitors.”
  Please, rephrase to “However, PAR1 inhibitors did not rescued the barrier dysfunction after long-term co-culture with IE lysate”. The molecular mechanisms has not been described in this manuscript.

Reproducibility of Methods:

- Which amount of IE is used in the long-term co-culture in Figures 3, 4 and 5? The authors give the parasitemia, the hematocrit but as the volume is missing, the quantity or concentration of IE cannot be calculated.  

Results:

- Why did the authors test the rescue after short-term EPCR-PfEMP1 steric inhibition on EA.hy.926 cells (HUVEC) (behavior A) and the long-term parasite effects on HBMEC (behavior B)? Is the rescue seen on Figure 2 also reproducible on HBMEC?

Figures and figure legends:

- A visual timeline of the experiments in each figure might improve the clarity. The experiments performed are quite complex and difficult to interpret and to compare with the current figure legends.  

References:

- Kessler *et al.*, 2017 is not well cited in the first paragraph as a review of aPC-PAR1 axis. Is this a typo?
- In the current manuscript Avril *et al*., 2019 is only being cited in the introduction along with 5 other papers. The paper should be introduced in the result section “ *Behaviour B has been reported previously by several investigators using a range of co-culture systems (* *Tripathi et al., 2007* *) ( Gillrie et al., 2012* *), with either IE or parasite material affecting barrier integrity*.” Given the similarities and complementarity between Storm *et al.,* 2020 and Avril *et al.*, 2019, it might be beneficial to discuss it more extensively in the discussion.

Is the work clearly and accurately presented and does it cite the current literature?

Partly

If applicable, is the statistical analysis and its interpretation appropriate?

Yes

Are all the source data underlying the results available to ensure full reproducibility?

Yes

Is the study design appropriate and is the work technically sound?

Yes

Are the conclusions drawn adequately supported by the results?

Yes

Are sufficient details of methods and analysis provided to allow replication by others?

Partly

Reviewer Expertise:

Malaria pathogenesis, host-parasite interactions, vascular biology and engineering.

I confirm that I have read this submission and believe that I have an appropriate level of expertise to confirm that it is of an acceptable scientific standard.

# Response

**Published:** 2020-05-19

**Authors:** Janet Storm

**Affiliations:** Liverpool School of Tropical Medicine, UK

We thank the reviewer for her constructive comments and have addressed these in our reply and in the manuscript.

**The authors used the same parasitemia and hematocrit for long-term culture or to prepare the IEs lysate and they obtained significantly differences in barrier dysfunction. Did the authors observe merozoite egress after long term IE incubation?**

In similar co-culture experiments (in EC culture medium) the parasites are still healthy looking after 6-7 hours. However, after overnight co-culture they do not develop properly and we hardly ever saw newly formed rings. In using IE, we are specifically looking at the effect of adhesion rather than the release of parasite material.

**Are the molecules released on a mid-trophozoite lysate a good proxy of schizont molecules released after rupture** *?*

To mimic the release of schizont molecules, we wanted to use lysate of schizonts and we spent considerable time optimising culture methods to obtain parasites in schizogony. This proved to be rather difficult and therefore we made lysates of trophozoites in a late as possible stage. The cultures were regularly synchronised with sorbitol to keep them synchronous. In the methods section, preparation of IE and lysates has been altered to mid-late trophozoite stage. We did notice that lysates with a higher proportion of schizonts had a greater effect on the cell index, but for the experiments described in this manuscript it was not investigated further.

**Is the concentration used “physiologically” present in the cerebral malaria microvasculature. Although I agree that the concentration used in this study might be necessary to observe a major barrier breakdown in in vitro cultures, it might not be accurately mimic the pathogenic mechanisms on patients. A sentence in the discussion reflecting the limitations of the model might improve the interpretation of the paper by readers outside the field.**

The reviewer is correct in pointing out that the *in vitro* conditions might not mimic the *in vivo* situation accurately, but the release of parasite molecules at sequestration sites could result in high local concentrations of these parasites factors. Measurements from patient plasma may only partially reflect these local concentrations, but hopefully new 3D models of cytoadherence may be able to resolve this issue. A comment has been added to the discussion to reflect this.

**Long term incubation with both IE or IE lysate showed 1) reduced surface expression of EPCR and reduced HBMEC cell index. However, the reduced cell index might not be a direct consequence of loss of EPCR in the surface. As the authors accurately describe in the discussion, loss of barrier in their experiments is more likely a consequence of the presence of parasite histones, HRP2 or other soluble mediators. However, the hypothesis exposed in the introduction might be misleading for the average reader. *“Our hypothesis is that, through both of these mechanisms, the Thrombin-PAR1 coagulation/inflammation axis plays a significant role in barrier loss in CM, and that adjunct treatments targeting this might alleviate mortality or post-CM neurological sequelae in CM. Since the activation of PC may be prevented by either of these mechanisms of EPCR disruption (steric inhibition or receptor cleavage) it is important to identify treatments that could be barrier stabilising to EPCR abrogation by either mechanism.”* Since we don’t understand quite well the molecular mechanisms that induce loss of endothelial integrity and surface EPCR, the authors should be more careful while establishing associations.**

The reason to test the PAR1 inhibitors was to test our initial hypothesis that the Thrombin-PAR1 axis plays a significant role in barrier loss in CM. If true, the PAR1 inhibitors should affect the parasite-induced disruption of barrier function.  Our results show that this is not the case and the implications of these data are discussed. We have changed the wording at the start of this paragraph to indicate the context of the hypothesis as a starting point. We agree with the reviewer that we do not understand all the mechanisms that contribute to endothelial dysfunction in severe malaria.

**I would recommend to include both 1) representative traces of cell index and bar plots in figure 3, 4, 5 and 6 in a similar way than in figure 2. Or include it in supplementary data.**

Cell index traces have been added to figure 5 and 6. In figure 5B, the plot shows the effect of Vorapaxar on thrombin induced disruption of barrier function in the absence and presence of lysate. Figure 5A depicts the same traces but over a longer time frame, also showing the effect of Vorapaxar on IE lysate induced decrease in cell index. Figure 6A then shows the effect of all four inhibitors on the lysate induced decrease in cell index.

Note that the calculations are also provided in the raw data files “Combined analysis data for fig5_TEER_includes normalisation_2020-02-04.csv” and “Combined analysis data for fig6_TEER_includes normalisation_2020-02-04.csv”.

Figure 4 already depicts TEER traces, but a panel (4B) has been added on request from both reviewers. It shows that HBMEC exposed to IE lysate recovers slowly from thrombin induced barrier disruption.

Figure 3 shows the EPCR expression data, derived from flow cytometry.

**Was the experiment shown in figure 4 done on independent days?**

Yes, the data shown in figure 4 A, B and C (note that there is an additional panel) are from three different experiments.

**Although TEER measurements are highly variable depending on the day of the experiment, box plots and dot plots might better represent data distribution than bar graphs.**

The bar graphs in figure 5 and 6 are showing mean ± SD, with relatively low SD values. We do not think depicting it as box plots or dot plots will change the interpretation of the data.

**Avril *et al.*, 2019 showed using the same methodology that schizont-stage parasites (EPCR binding strains) prolonged thrombin-induced barrier disruption in resting and activated HBMEC, and HBMEC do not get back to baseline after IE incubation. Thrombin and IE colocalization is frequent in the microvasculature in cerebral malaria autopsies. As the effects seen in Avril *et al.* are likely to occur in patients this experiment is highly relevant. Is the experiment in Figure 5 equivalent to Avril *et al.*, 2019 but with trophozoites lysates expressing CD36-binding PfEMP1? At which time-scale was the CI measured in figure 5? A representative cell index trace in Figure 5 will clarify that.**

We thank the reviewer for their comment and we observed the same delayed recovery from thrombin treatment after co-culture with IE lysate. We have added a representative cell index trace to show this in figure 5 (A and B) and added text to the results and discussion sections to acknowledge this.

A similar cell index trace was also added to figure 4 to show all three observations with HBMEC in one figure:

A) Barrier function disruption by thrombin with a fast recovery. This can partly be rescued by the addition of aPC.

B) Slow recovery from thrombin treatment after co-culture with IE lysate.

C) Disruption of barrier function by IE lysate.

**“However, in the IE lysate co-culture system we identified a mechanism that slowly reduces barrier function and which is insensitive to PAR1 inhibitors.”**

**Please, rephrase to “However, PAR1 inhibitors did not rescued the barrier dysfunction after long-term co-culture with IE lysate”. The molecular mechanisms has not been described in this manuscript.**

The reviewer is correct and we have changed this in the abstract.

**Which amount of IE is used in the long-term co-culture in Figures 3, 4 and 5? The authors give the parasitemia, the hematocrit but as the volume is missing, the quantity or concentration of IE cannot be calculated.**

This has been added in the methods, it is 300 µl. The number of IE (or equivalent for lysates) has also been added, as requested by the second reviewer.

**Why did the authors test the rescue after short-term EPCR-PfEMP1 steric inhibition on EA.hy.926 cells (HUVEC) (behavior A) and the long-term parasite effects on HBMEC (behavior B)? Is the rescue seen on Figure 2 also reproducible on HBMEC?**

The initial screening experiments were done with a cell line to simplify the approach, and compounds that were identified from this screen were entered into more complex investigations using primary human brain endothelial cells.  We recognise that the screen may miss some details but we used this as a cost-effective way to down-select the number of experiments needed.  As others have reported on the steric effects of PfEMP1, we did not replicate this work on HBMEC. 

**A visual timeline of the experiments in each figure might improve the clarity. The experiments performed are quite complex and difficult to interpret and to compare with the current figure legends.**

A timeline has been added to figure 2, 4, 5 and 6.

**Kessler et al., 2017 is not well cited in the first paragraph as a review of aPC-PAR1 axis. Is this a typo?**

We had included it to give full coverage but the reviewer is correct and we have removed the reference at this point – it is cited later.

**In the current manuscript Avril et al., 2019 is only being cited in the introduction along with 5 other papers. The paper should be introduced in the result section *“Behaviour B has been reported previously by several investigators using a range of co-culture*** ***systems ( Tripathi et al., 2007) ( Gillrie et al., 2012), with either IE or parasite material affecting*** ***barrier integrity.”*** **Given the similarities and complementarity between Storm et al., 2020 and Avril et al., 2019, it might be beneficial to discuss it more extensively in the discussion.**

The reference to *Avril et al* has been added to the results and discussion section, specifically after adding TEER traces to figure 4 and 5 describing a similar observation with delayed thrombin recovery after IE lysate exposure.
